# Therapeutic Effect of a Poly(ADP-Ribose) Polymerase-1 Inhibitor on Experimental Arthritis by Downregulating Inflammation and Th1 Response

**DOI:** 10.1371/journal.pone.0001071

**Published:** 2007-10-31

**Authors:** Elena Gonzalez-Rey, Ruben Martínez-Romero, Francisco O'Valle, Rocío Aguilar-Quesada, Carmen Conde, Mario Delgado, F. Javier Oliver

**Affiliations:** 1 Institute of Parasitology and Biomedicine, Consejo Superior de Investigaciones Científicas (CSIC), Granada, Spain; 2 Department of Pathology, School of Medicine, Granada University, Granada, Spain; 3 Research Laboratory, Hospital Clinico Universitario, Santiago de Compostela, Spain; Université de Toulouse, France

## Abstract

Poly(ADP-ribose) polymerase-1 (PARP-1) synthesizes and transfers ADP ribose polymers to target proteins, and regulates DNA repair and genomic integrity maintenance. PARP-1 also plays a crucial role in the progression of the inflammatory response, and its inhibition confers protection in several models of inflammatory disorders. Here, we investigate the impact of a selective PARP-1 inhibitor in experimental arthritis. PARP-1 inhibition with 5-aminoisoquinolinone (AIQ) significantly reduces incidence and severity of established collagen-induced arthritis, completely abrogating joint swelling and destruction of cartilage and bone. The therapeutic effect of AIQ is associated with a striking reduction of the two deleterious components of the disease, i.e. the Th1-driven autoimmune and inflammatory responses. AIQ downregulates the production of various inflammatory cytokines and chemokines, decreases the antigen-specific Th1-cell expansion, and induces the production of the anti-inflammatory cytokine IL-10. Our results provide evidence of the contribution of PARP-1 to the progression of arthritis and identify this protein as a potential therapeutic target for the treatment of rheumatoid arthritis.

## Introduction

Rheumatoid arthritis (RA) is an autoimmune disease that leads to chronic inflammation in the joints and subsequent destruction of the cartilage and erosion of the bone. Although its etiology is unknown, evidences indicate that the recruitment and activation of neutrophils, macrophages and lymphocytes into joint tissues and the formation of the pannus are hallmarks of the pathogenesis of RA. Although the contribution of Th1 responses in RA is not completely understood, several studies in animal models point to a pathogenic role for Th1-derived cytokines [1]–[3]. Th1 cells reactive to components of the joint, infiltrate the synovium, release proinflammatory cytokines and chemokines, and promote macrophage and neutrophil infiltration and activation. Inflammatory mediators, such as cytokines and free radicals, produced by infiltrating inflammatory cells, play a critical role in joint damage [3], [4]. The fact that the inflammatory process in RA is chronic suggests that immune regulation in the joint is disturbed. This disturbance is probably caused by an excessive inflammatory response together with a deficiency in the mechanisms that control the immune response. Available therapies based on immunosuppressive agents inhibit the inflammatory component of RA and have the potential to slow progressive clinical disability by delaying erosions and deformity (5,6). However, they neither reduce the relapse rate nor delay disease onset, and because a continued treatment is required to maintain a beneficial effect, they have multiple side effects [5], [6]. This illustrates the need for novel therapeutic approaches to prevent the inflammatory and autoimmune components of the disease and to promote immune tolerance restoration.

Poly(ADP-ribose) polymerase-1 (PARP-1) is a nuclear DNA-binding protein activated by DNA damage, belonging to a family of more than seventeen members, that catalyzes the attachment of ADP-ribose to target proteins, acts as a component of enhancer/promoter regulatory complexes and participates in the regulation of DNA repair and genomic integrity maintenance [7]. Numerous studies have also involved PARP-1 in the regulation of the inflammatory response [8]. Thus, animals treated with PARP-1 inhibitors or PARP-1 deficient mice showed decreased tissue damage and inflammatory mediators in several models of ischemia-reperfusion and heart transplantation (reviewed in [9]). Moreover, mice lacking PARP-1 are extremely resistant to endotoxin-induced septic shock [10]. The involvement of PARP-1 in the pathogenesis of autoimmune disorders has been previously suggested. Thus, PARP-1 inhibition prevented progression of type 1 diabetes, inflammatory bowel disease and experimental autoimmune encephalomyelitis [11]–[14]. Interestingly, a promoter haplotype for PARP-1 has been shown to confer higher susceptibility for systemic lupus erythematous and RA [15], [16]. Therefore, the aim of this study is to investigate the potential impact of selective PARP-1 inhibition in an experimental model of RA. Here we show that delayed treatment with a novel PARP-1 inhibitor has great benefit at the clinical and pathological levels, as its therapeutic action was exerted at multiple levels, being associated with the downregulation of inflammatory and Th1-mediated autoimmune components of the disease. These results provide support for the contribution of PARP-1 in the pathogenesis of arthritis and open the possibility that specific PARP-1 inhibitors might become attractive therapeutic tools in RA.

## Results

### PARP-1 inhibition decreases severity of experimental arthritis

**Figure 1:**
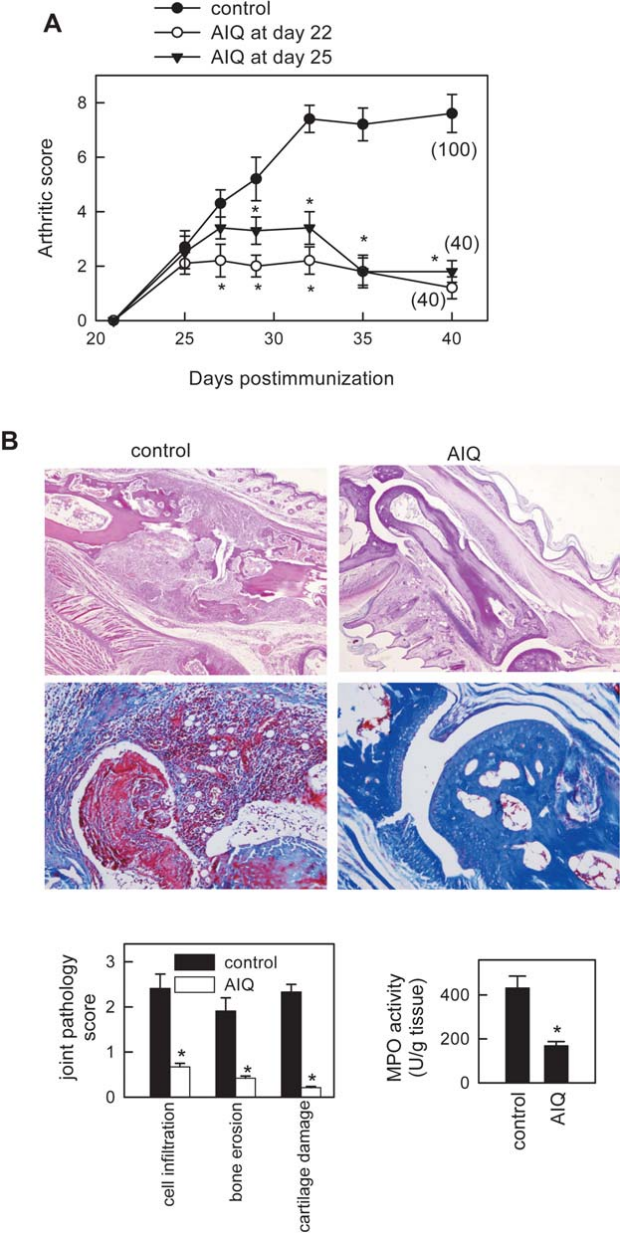
AIQ decreases CIA severity. DBA1 mice with established CIA were injected i.p. either with PBS (control) or with AIQ (1.5 mg/kg) on day 22 or on day 25. A. Severity of arthritis was assessed by clinical scoring. Numbers in parenthesis represent frequency of arthritis (% mice with arthritis score>2 at day 35). B. Histological analysis of trichromic-stained (lower) or H&E-stained (upper) sections of joints obtained at day 45 was performed. Scoring of inflammation, cartilage damage and bone erosion of paws from untreated (control) and AIQ-treated CIA mice is shown. Neutrophil infiltration in the joints was determined by measuring MPO activity in protein extracts isolated at day 35. n = 6–8 mice per group. *p<0.001 versus control.

Collagen-induced arthritis (CIA) is a murine experimental disease model that shares a number of clinical, histologic and immunologic features with RA, and it is used as a model system to test potential therapeutic agents. 5-aminoisoquinolinone (AIQ) is a new and selective inhibitor of PARP-1 previously used for the treatment of various ischemic/reperfusion injuries [17], [18]. A single administration of AIQ (1.5 mg/kg) at the onset of the disease or to mice with established clinical signs of arthritis progressively attenuated the severity of CIA and decreased the percentage of mice with arthritis, as compared to untreated mice (Fig. 1A). We did not observe significant differences in the therapeutic effect of AIQ at 1.5 mg/kg and 3 mg/kg doses (not shown). Histopathological analyses of joints showed that AIQ treatment significantly abrogated CIA-characteristic chronic inflammation of synovial tissue (infiltration of inflammatory cells into the joint cavity and periarticular soft tissue, consisting in lymphocytes, plasma cells, macrophages and neutrophils), pannus formation, cartilage destruction and bone erosion (Fig. 1B). The AIQ-mediated inhibition of neutrophil infiltration was confirmed with decreased joint myeloperoxidase (MPO) activity (Fig. 1B).

### AIQ inhibits inflammatory response in CIA

**Figure 2:**
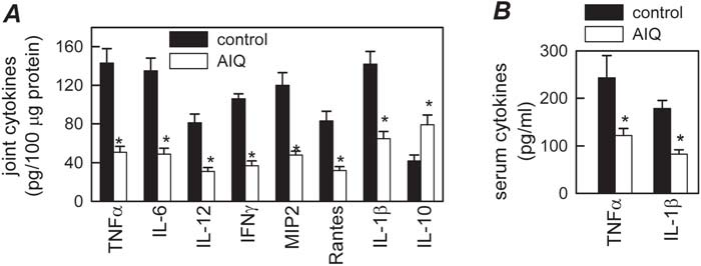
AIQ administration inhibits inflammatory response in CIA. DBA1 mice with established CIA were injected i.p. either with PBS (control) or with AIQ (1.5 mg/kg) on day 25 post-immunization. Systemic and local expression of inflammatory mediators was assayed in protein extracts from joints (A) and sera (B) isolated at day 35 post-immunization. A paw from an unimmunized mouse was analyzed simultaneously for assessment of the basal response. n = 3–4 mice/group. *p<0.001 versus controls.

We next investigated the mechanisms underlying the decrease in severity of CIA following PARP-1 inhibition. Several evidences have involved a wide array of cytokines and chemokines in joint inflammation and the arthritis progression [1], [3], [19]. We evaluated the effect of AIQ treatment on the production of inflammatory mediators that are mechanistically linked to CIA severity. AIQ administration significantly reduced protein and gene expression of inflammatory cytokines (TNFα, IFNγ, IL-6, IL-1β and IL-12), chemokines (Rantes and MIP-2) in the joint of arthritic mice (Fig. 2A). In addition, joints of AIQ-treated mice showed increased levels of the anti-inflammatory cytokine IL-10 (Fig. 2A). The broad anti-inflammatory activity of AIQ in the inflamed joint was accompanied by downregulation of the systemic inflammatory response. In vivo inhibition of PARP-1 decreased CIA-induced serum levels of the proinflammatory cytokines TNFα and IL-1β (Fig. 2B).

### In vivo inhibition of PARP-1 downregulates Th1-mediated CII-specific response in CIA

**Figure 3:**
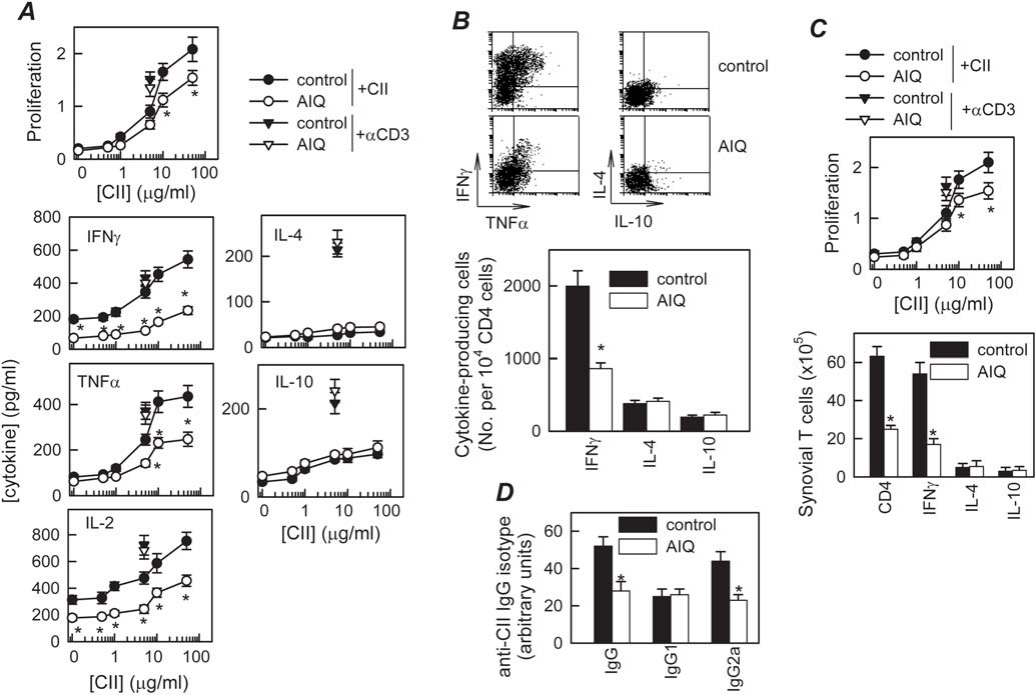
Inhibition of PARP-1 downregulates Th1-mediated response in CIA. DBA1 mice with established CIA were injected i.p. either with PBS (control) or with AIQ (1.5 mg/kg) on day 25 post-immunization. A. Proliferative response and cytokine production of DLN cells isolated at day 30 from untreated (control) or AIQ-treated CIA mice were determined after in vitro stimulation with different concentrations of CII. Stimulation of DLN cells with anti-CD3 antibodies (▾, for untreated CIA mice; ▿, for AIQ-treated CIA mice) was used for assessment of nonspecific stimulation. A pool of 3 nonimmunized DBA/1 DLN cells was used for assessment of the basal response. No proliferation or cytokine production by T cells was detectable in the presence of an unrelated antigen (OVA). n = 5 mice/group. B, Number of CII-specific cytokine-producing T cells. DLN cells from untreated (control) or AIQ-treated CIA mice were restimulated in vitro with CII (10 µg/ml) and analyzed for CD4 and intracellular cytokine expression in gated CD4 T cells by flow cytometry. Dot plots show representative double staining for IFNγ/TNFα or IL-4/IL-10 expression in gated CD4 T cells. The number of IFNγ-, IL-4- and IL-10-expressing T cells relative to 10^4^ CD4 T cells is shown in the lower panel. Data shown represent pooled values from two independent experiments. C, CII-specific proliferative response and the number of cytokine-producing CD4 T cells were determined in synovial membrane cells isolated from untreated (control) or AIQ-treated CIA mice and stimulated in vitro with CII (10 µg/ml) for 48 h. Data show the results of pooled synovial cells from 3 animals per group. D. AIQ decreases titter of autoantigens in CIA mice. The levels of CII-specific IgG, IgG1 and IgG2a antibodies in sera collected at day 35 were determined by ELISA. n = 3-5 mice/group. *p<0.001 versus controls.

Although macrophages and neutrophils are the major sources of inflammatory mediators, CD4 T cells play a key role in the initiation and perpetuation of CIA by producing IFNγ, a potent inducer of the inflammatory response. In fact, CIA is considered an archetypal of Th1-type cell-mediated autoimmune disease [1]. Therefore, PARP-1 inhibition could ameliorate CIA by reducing autoreactive T-cell responses and/or migration to the joints. We determined proliferation and cytokine profile of draining lymph node (DLN) cells isolated from AIQ-treated arthritic mice in response to antigen (CII) in vitro. DLN cells obtained from CIA mice showed marked CII-specific proliferation and effector T cells producing high levels of Th1-type cytokines (IFN-γ, IL-2 and TNF-α) and low levels of Th2-type cytokines (IL-4 and IL-10) (Fig. 3A). In contrast, DLN cells from AIQ-treated mice proliferated much less, produced low levels of Th1 cytokines (Fig. 3A). The Th2-type cytokines IL-4 and IL-10 were not significantly affected (Fig. 3A). This effect was antigen-specific, because AIQ treatment did not affect proliferation and cytokine production by anti-CD3-stimulated spleen cells (Fig. 3A). This suggests that inhibition of PARP-1 during CIA progression partially inhibits CII-specific Th1-cell clonal expansion. In order to distinguish whether the decrease in Th1 cytokine production induced by AIQ treatment is consequence of either downregulation of cytokine release or inhibition of Th1 cell expansion, we determined the intracellular expression of these cytokines by flow cytometry in sorted CD4 T cells. AIQ significantly decreased the number of IFNγ-producing Th1 cells, although did not affect the number of IL-4/IL-10-producing CD4 T cells in DLN (Fig. 3B). We observed similar effects on synovial cells (Fig. 3C). Thus, in vivo inhibition of PARP-1 on CIA mice regulates the expansion of autoreactive/inflammatory Th1 cells.

High levels of circulating antibodies directed against collagen rich joint tissue invariably accompany the development of RA and CIA, and their production is a major factor in determining susceptibility to the disease [20]. AIQ administration resulted in reduced serum levels of CII-specific IgG, particularly autoreactive IgG2a antibodies (Fig. 3D), generally reflective of Th1 activity. These data provide further evidence that PARP-1 inhibition during CIA reduces the Th1 autoreactive responses both in the joint and periphery.

## Discussion

The initial stages of RA and CIA involve multiple steps, which can be divided into two main phases: initiation and establishment of autoimmunity, and later events associated with the evolving immune and inflammatory responses. The crucial process underlying disease initiation is the induction of autoimmunity to collagen rich joint components; later events involve a destructive inflammatory process [1], [3]. Progression of the autoimmune response involves the development of autoreactive Th1 cells, their entry into the joint tissues, and future recruitment of inflammatory cells through multiple mediators. Certain therapeutic approaches address the autoimmune component of CIA and RA, complementing existing anti-inflammatory therapies. In this study we show that a specific and potent inhibitor for PARP-1 provides a highly effective therapy for CIA. The therapeutic effect of AIQ is associated with a striking reduction of the two deleterious components of the disease, i.e. the autoimmune and inflammatory response. In vivo inhibition of PARP-1 decreased the presence of autoreactive Th1 cells in the periphery and the joint. In addition, AIQ strongly reduced the inflammatory response during CIA progression by downregulating the production of several inflammatory mediators, such as various cytokines and chemokines in the joints. As a consequence, treatment with AIQ reduced the frequency of arthritis, ameliorated symptoms and avoided joint damage. From a therapeutic point of view, it is important to take in account the ability of delayed administration of AIQ to ameliorate ongoing disease, which fulfills an essential prerequisite for an anti-arthritic agent, as treatment is started after the onset of patient arthritis. The fact that we did not observe a loss in its beneficial effect with time suggests that an initial treatment with AIQ could induce remission of the disease. Therefore, a long-term treatment may not be required with AIQ, avoiding the appearance of potential side effects. AIQ offers therapeutic advantages over other PARP inhibitors, such as benzamide and the phenanthridinone derivate PJ34, used in several autoimmune models [11]–[14]. Thus, PJ34 showed a protective effect on experimental autoimmune encephalomyelitis, CIA and experimental type 1 diabetes only when administered on a prophylactic regime before the disease onset, loosing its therapeutic effect in animals with established clinical signs [13]. In addition, contrary to AIQ, the protective effects of these PARP-1 inhibitors disappeared once treatment is terminated [11]–[14].

The capacity of AIQ to regulate a wide spectrum of inflammatory mediators might offer a therapeutic advantage over other treatments directed against a single mediator, such as the new biologic agents. Chemokines are responsible for the infiltration into the joint and activation of various leukocyte populations, which contribute to CIA pathology (1-3,[21]. The fact that in vivo inhibition of PARP-1 reduced the expression of a plethora of chemokines could partially explain the absence of inflammatory infiltrates in the joint tissues of AIQ-treated mice, being especially relevant for chemokines as MIP-2 (chemotactic for neutrophils) and Rantes (for macrophages and T cells), all involved in CIA pathogenesis [21], [22]. Moreover, PARP-1 inhibition reduced the expression of several proinflammatory chemokines receptors involved in arthritis pathology (not shown), reflecting also a decreased joint inflammatory infiltration.

In addition to the regulation of cell recruitment to the joint, AIQ also regulates the activation of inflammatory cells in the joints. Thus, AIQ downregulated the production of the proinflammatory/cytotoxic cytokines TNFα, IFNγ, IL-6, IL-1β and IL-12 in the inflamed joint, and increased the levels of the anti-inflammatory cytokine IL-10, which ameliorates the disease [23]. The decrease in inflammatory mediators could be the consequence of a diminished infiltration of inflammatory cells in the synovium. However, the fact that AIQ inhibited the production of pro-inflammatory mediators by synovial cells isolated from CIA mice, argues against this hypothesis (not shown). This suggests that, in addition to the reduction in inflammatory infiltration, the inhibition of PARP-1 deactivates the inflammatory response.

Two mechanisms have been proposed to explain the role of PARP-1 in inflammatory diseases. One potential mechanism is related to massive PARP-1 activation induced by genotoxic injury developed during the inflammatory process [24]. In this case, hyperactivated PARP-1 would lead to rapid ATP depletion and to irreversible cellular energy failure and necrotic-type cell death subsequent to disruption of oxidative metabolism. However, several lines of evidence suggest that under inflammatory conditions the beneficial effects of PARP-1 inhibition are independent on the prevention of energy failure (12). The “suicide hypothesis”, therefore, might be valid only in conditions of massive DNA rupture and intense PARP-1 activation. The other proposed and more plausible mechanism is related to a functional link between PARP-1 and inflammation-related transcription factors. Several *in vivo* and *in vitro* studies have demonstrated the involvement of PARP-1 in the activation of nuclear factor NF-κB [10], [25], a transcription factor that plays a central role in the regulation of genes involved in the immune and inflammatory response of RA. Recently, it has also been reported that PARP-1 regulates other transcription factors implicated in stress/inflammation, such as AP-1, Oct-1, SP-1, YY-1 and Stat-1 [26], [27].

CIA is also a Th1-mediated disease, and the bias towards Th1 cytokines (mainly IFNγ and TNFα) is crucial in the establishment of chronic inflammation in the joint (1-3). Our results demonstrate that the administration of AIQ to arthritic mice results in a decreased CII-specific Th1-mediated response. It appears that the inhibition of the Th1 response is caused by a direct action on synovial and DLN cells, since synovial and DLN cells obtained from AIQ-treated animals are refractory to Th1 cell stimulation. Whereas the effects of PARP-1 inhibition on the inflammatory response have been previously suggested by others, the present study is the first evidence describing the involvement of PARP-1 in the Th1-mediated autoreactive response. Although the mechanisms involved in the inhibitory action of AIQ in Th1 activation are unknown, our results suggest that AIQ treatment impairs the proliferation and/or differentiation of antigen-specific Th1 cells. In this sense, NF-kB and AP-1 are involved in the regulation of the proliferative response of T cells, and AIQ inhibition of PARP-1-mediated activation of these transcription factors could play a major role in this effect. Indeed, other PARP-1 inhibitors have been found to decrease NF-kB and AP-1 transduction activity in activated T cells [11].

In summary, this work identifies PARP-1 as a novel therapeutic target for the treatment of RA and other chronic autoimmune disorders and provides a powerful rationale for the assessment of the efficacy of the PARP-1 inhibitor AIQ as a new immunomodulatory factor with the capacity to deactivate the inflammatory response in vivo at multiple levels.

## Methods

### Arthritis induction and treatment

Animal experimental protocols were reviewed and approved by the Ethical Committee of the Spanish Council of Scientific Research (CSIC). To induce collagen-induced arthritis (CIA), DBA/1J mice (7–10-wk-old, Jackson Labs) were injected s.c. with 200 µg of type II collagen (CII, Sigma) emulsified in complete Freund́s adjuvant (CFA) containing 200 µg of *M. tuberculosis* H37 RA (Difco, Detroit, Michigan). At day 20 after primary immunization, mice were boosted s.c. with 100 µg of CII in CFA. Treatment with PARP-1 inhibitor (AIQ, Alexis) consisted in a single administration i.p. of 1.5 mg/kg of AIQ (in saline) starting at the disease onset (at day 22 post-immunization) or at 25 days post-immunization when all mice showed established arthritis (clinical score>2). The dose of AIQ was selected from previous in vivo studies demonstrating the efficacy and potency of the drug to inhibit PARP-1 activity without any resultant toxicity [17], [18]. In each experiment, a control group of mice was injected i.p. with PBS (untreated). Mice were analyzed by two independent, blinded examiners every other day and scored for signs of arthritis by using the following system: grade 0, no swelling; grade 1, slight swelling and erythema; grade 2, moderate swelling and edema; grade 3, extreme swelling and pronounced edema; grade 4, joint rigidity. Each limb was graded, giving a maximum possible score of 16 per animal.

### Histopathology analysis

For histological analysis, the paws were randomly collected by two independent experimenters at day 45 after primary immunization, fixed in 4% buffered-formaldehyde, decalcified, paraffin-embedded, sectioned and stained with H&E or Masson-Goldner trichromic stain. Histopathological changes were scored in a blinded manner based in cell infiltration, cartilage destruction and bone erosion parameters as described [28]. Neutrophil infiltration in the joints was monitored by measuring myeloperoxidase (MPO) activity in joint extracts isolated at day 35 post-immunization as described [28].

### Cytokine and autoantibody determination

For cytokine determination in joints, protein extracts were isolated by homogenization of joints (50 mg tissue/ml) in 50 mM Tris-HCl, pH 7.4, with 0.5 mM DTT, and proteinase inhibitor cocktail (10 µg/ml, Sigma). Serum samples were collected at peak of disease (day 35) and the levels of anti-CII IgG, IgG1 and IgG2a Abs were measured by ELISA as described [29]. Cytokine and chemokine levels in the serum and joint protein extracts prepared at the disease peak (day 35) were determined by specific sandwich ELISAs using capture/biotinylated detection Abs from BD Pharmingen (San Diego, CA) according to the manufacture's recommendations.

### Assessment of T cell autoreactive response

Because T cell autoreactive response precedes to maximal clinical manifestations of the disease, single-cell suspensions (10^6^ cells/ml) from draining lymph nodes (DLN) and synovial membrane of knee joints were obtained at 30 days post-immunization. Cells were stimulated in complete medium (RPMI 1640 containing 10% FCS, 2 mM L-glutamine, 100 U/ml penicillin, and 100 µg/ml streptomycin) with different concentrations of heat-inactivated CII for 48 h (for cytokine determination) or for 72 h (for proliferative response) [29]. Cell proliferation was evaluated by using a cell proliferation assay (BrdU) from Roche Diagnostics GmbH (Mannheim, Germany). Cytokine content in culture supernatants was determined by specific sandwich ELISAs as above. For intracellular analysis of cytokines, DLN and synovial cells were stimulated with inactivated CII (10 µg/ml) for 8 h, in the presence of monensin, and then stained with PerCP-anti-CD4 mAbs at 4°C, washed, fixed/saponin permeabilized, stained with FITC- and PE-conjugated anti-cytokine specific mAbs (BD Pharmingen), and analyzed on a FACScalibur flow cytometer (Becton Dickinson). To distinguish between monocyte/macrophage and T cell sources, intracellular cytokine analysis was done exclusively in the PerCP-labeled CD4 T cell population.

### Data analysis

All values are expressed as mean±SD. The differences between groups were analyzed by Mann-Whitney U test and, if appropriate, by Kruskal-Wallis ANOVA test.
